# Knowledge and practice of surgical hand hygiene of healthcare worker at University Medical Center, Ho Chi Minh City, 2022

**DOI:** 10.1017/ash.2025.143

**Published:** 2025-09-03

**Authors:** Truong Thi Le Huyen, Pham Thi Lan, Tran Nguyen Giang Huong, Nguyen Thi Minh Khai, Le Thanh Truyen, Huynh Hoang Hai, Trinh Thi Thoa, Nguyen Vu Hoang Yen, Huynh Minh Tuan

**Affiliations:** 1 University Medical Center, HCM city; 2 University of Medicine and Pharmacy at HCM city

**Keywords:** Surgical hand hygiene, Surgical hand hygiene procedure, hand hygiene

## Abstract

**Introduction:** Nowadays, surgical site infection is one of the four common types of healthcare-associated infections. There are many preventive measures applied and surgical hand hygiene (SHH) is the most effective and the simplest measure. This study aimed to assess the knowledge, practices of SHH among staff and the relationship between knowledge and practice of SHH in Viet Nam. **Methods:** An analytical cross-sectional study was conducted at the University Medical Center (UMC) in Ho Chi Minh City in 2022. The study employed a set of pre-prepared questions for the knowledge assessment section. For the practical assessment section, the research team conducted indirect observation through cameras and filled out a monitoring checklist. The data were analyzed using Stata 13.2. **Results:** Of the 271 healthcare workers, surgeons had the highest proportion at 48.7%, which was 18.6 times higher than that of anesthesiologists. The majority of healthcare workers received training on SHH, accounting for a rate of 95.6%. Among the participants, the overall compliance of SHH before entering the operative room accounted for 85.6%. The percentage of correct general knowledge reached 73.8%, and there was a relationship between correct knowledge and correct practice with p < 0.01. **Conclusion:** Our data suggests that having correct general knowledge of SHH is a crucial factor in accurately practicing SHH. Therefore, providing training to impart accurate knowledge about SHH to healthcare staff is necessary to enhance the overall compliance rate of SHH before entering the operating room.

